# Outcomes of Combined Left Atrial Appendage Occlusion and Transcatheter Mitral Edge-to-Edge Repair: The WATCH-TEER Study

**DOI:** 10.1016/j.jacadv.2024.101541

**Published:** 2025-01-08

**Authors:** Abdullah Al-Abcha, Pietro Di Santo, Charanjit S. Rihal, Trevor Simard, Benjamin Hibbert, Mohamad Alkhouli

**Affiliations:** a Department of Cardiovascular Medicine, Mayo Clinic College of Medicine and Science, Rochester, Minnesota, USA; b Division of Cardiology, Department of Medicine, University of Ottawa, Ottawa, Ontario, Canada

**Keywords:** atrial fibrillation, left atrial appendage occlusion, mitral regurgitation, transcatheter mitral edge-to-edge repair

## Abstract

**Background:**

Up to 50% of patients undergoing mitral transcatheter edge-to-edge repair (MTEER) have an indication for left atrial appendage occlusion (LAAO). However, prospective evaluation of this strategy is lacking.

**Objectives:**

The aim of the study was to prospectively evaluate the outcomes of combined LAAO and MTEER.

**Methods:**

The WATCH-TEER study is a prospective multicenter registry that aims to assess the feasibility and safety of concomitant MTEER with MitraClip and LAAO with WATCHMAN-FLX in patients with an approved clinical indication for both procedures. The primary endpoint was a composite of all-cause mortality, stroke, life-threatening, or major bleeding at 45 days.

**Results:**

A total of 24 patients were included between October 2020 and March 2024. Mean age was 79.5 ± 6.3 years, and 83% were males. The Society of Thoracic Surgeons operative risk score was 11.8% ± 5.3%, the CHA2DS2-VASc score was 4.5 ± 1.1, and the HAS-BLED score was 3.3 ± 1.5. Total procedure time was 103.6 ± 33.7 minutes. At 45 days, the primary endpoint occurred in 21% (95% CI: 5%-37%, n = 5/24) of patients, all of which occurred after discharge including 1 cardiac death, 1 ischemic stroke, 1 trauma-related intracranial hemorrhage, and 2 nonprocedural major bleeds. At 45 days, most patients (68%) had ≤2+ mitral regurgitation, and 72% of patients were in NYHA functional class I-II symptoms. Additionally, 71% of patients were not on anticoagulation, compared with only 20% at baseline.

**Conclusions:**

Combining LAAO with MTEER is feasible in patients who have a clinical indication for both procedures.

Mitral regurgitation (MR) is the second most common valve disease in the United States.^1^, ^2^, ^3^ Significant MR is present in up to 40% of patients admitted for heart failure (HF)^4^ and is associated with increased morbidity and mortality.^5^ Transcatheter mitral edge-to-edge repair (MTEER) is approved for the treatment of patients with severe degenerative MR who are deemed high risk for surgery and patients with severe symptomatic functional MR despite optimal medical therapy.^6^^,^^7^

Atrial fibrillation (AF) is the most common arrhythmia in older individuals and is common among patients referred for MTEER. Oral anticoagulation (OAC) is the mainstream strategy for most AF patients to mitigate their increased risk of stroke. However, a large proportion of patients either discontinue or have contraindication to OAC.^8^ To address this need, left atrial appendage occlusion (LAAO) has emerged as a feasible alternative to OAC in selected patients with nonvalvular AF.^9^^,^^10^

Combining clinically indicated structural heart interventions has potential logistical and clinical benefits. Hence, this concept has been discussed extensively in the literature with a few studies designed specifically to address its feasibility, safety, and clinical utility.^11^^,^^12^ The value of this “1-stop shop” approach may be more plausible in patients undergoing left-sided interventions.^13^, ^14^, ^15^, ^16^, ^17^ Both MTEER and LAAO require large-bore venous access, transseptal catheterization, and echocardiographic imaging guidance. Combining the 2 procedures in a single setting in patients who are suitable candidates for both may provide incremental logistical and clinical benefits. Few studies reported combined MTEER with LAAO, but prospective evaluation of this strategy is lacking.^18^, ^19^, ^20^ The WATCH-TEER study aims to prospectively assess the feasibility and safety of combining clinically indicated MTEER and LAAO at 2 tertiary centers.

## Methods

### Study design

The WATCH-TEER study is a prospective, multicenter registry that aims to assess the feasibility and safety of concomitant MTEER with the MitraClip device (Abbott) and LAAO with the WATCHMAN FLX device (Boston Scientific) in patients who have an approved clinical indication for both procedures. The study was registered at ClinicalTrials.gov (NCT04494347). Institutional review board approval was obtained at each participating WATCH-TEER trial site. The study was initially named WATCH-TMVr, but for consistency with current terminology, we will refer to it as WATCH-TEER throughout.

### Patient selection

Patients who were referred for MTEER, had a documented history of AF, and had an appropriate indication for LAAO were considered for enrollment in this trial. The trial’s detailed inclusion and exclusion criteria are reported in Supplemental Table 1.

### Study device and procedure

All patients underwent MTEER first under general anesthesia and transesophageal echocardiographic (TEE) guidance. At the conclusion of the MTEER procedure, patients underwent LAAO with the Watchman FLX device using the same transseptal access obtained to perform the MTEER procedure. Following the procedure, patients were discharged on either OAC with warfarin or a direct oral anticoagulant or dual antiplatelet therapy as per standard of care for 6 weeks. After 6 weeks, patients underwent TEE as per standard of care, an office visit, and complete clinical and neurological evaluation. Continuation or discontinuation of OAC depended on the 45-day TEE findings. Access site was closed with either figure of 8 suture or 2 Perclose ProGlide sutures (Abbott) at the discretion of the treating physician.

### Study endpoints

The primary endpoint was a composite of all-cause mortality, stroke, and life-threatening or major bleeding at 45 days. Secondary endpoints included all-cause mortality, cardiovascular mortality, ischemic or hemorrhagic stroke, major or life-threatening bleeding (VARC-2 definition), device embolization, residual MR, post-MTEER transmitral gradient, need for cardiac surgery, pericardial effusion, HF hospitalization, rehospitalization related to the LAAO device, and Watchman FLX device-related thrombus (DRT). These events were collected at 45 days and 1 year.

### Statistical analysis

Continuous variables are presented as mean ± SD or median (IQR). Categorical variables are presented as counts (percentages). Paired analysis for ordinal data was performed using the Wilcoxon signed rank test. Paired analysis comprised available data for the same patients across multiple specified timepoints. *P* value < 0.05 was considered statistically significant. All analyses were performed using R (version 4.3.1).

## Results

### Patients characteristics

A total of 25 patients were consented. One patient was excluded because LAAO was deferred after MTEER due to mitral valve injury and 24 patients were included between October 2020 and March 2024. Mean age was 79.5 ± 6.3 years, and 83% (20/24) of patients were males. All patients were symptomatic; 42% (10/24) of the patients had a history of HF hospitalization within 1 year prior to enrollment. Cardiovascular comorbidities were common (Table 1). All patients were at a high risk for stroke (CHA_2_DS_2_-VASc score of 4.5 ± 1.1) and bleeding (HAS-BLED score of 3.3 ± 1.5). A total of 17% had prior stroke, and 46% had a major bleeding event. Baseline echocardiography demonstrated that all patients had at least 3+ MR, and MR etiology was predominantly functional (67%). Leaflet tethering was present in 33% and mitral annular calcification in 38% (Table 2).

**Table 1:** Baseline Characteristics (N = 24)

Age, y	79.5 ± 6.3
Male	20 (83%)
Body mass index (kg/m^2^)	31.3 ± 8.1
Congestive heart failure	24 (100%)
Heart failure hospitalization within 1 y	10 (42%)
NYHA functional class III-IV	20 (83%)
Hypertension	25 (100%)
Previous stroke	4 (17%)
Chronic kidney disease, stage 3 or 4	19 (79%)
Hemodialysis	1 (4%)
Previous cardiac interventions	
PCI	10 (42%)
CABG	8 (33%)
TAVR	2 (8%)
SAVR	2 (8%)
TMVR	1 (4%)
PPM/ICD	10 (42%)
Frailty	9 (38%)
Major bleeding	11 (46%)
Intracerebral	1 (4%)
Gastrointestinal	7 (29%)
Other	5 (21%)
Thrombocytopenia	7 (29%)
Anemia	19 (79%)
STS PROM for MV replacement	11.8 ± 5.3
STS PROM for MV repair	8.1 ± 4.6
CHADSVASC score	2.8 ± 1.3
HAS-BLED score	3.3 ± 1.5
6-minute walk test	841.3 ± 319.3
Atrial fibrillation, pattern	
Paroxysmal	7 (29%)
Persistent	4 (17%)
Longstanding persistent	5 (21%)
Permanent	8 (32%)

Values are mean ± SD or n (%).

CABG = coronary artery bypass grafting; ICD = implantable cardioverter defibrillator; PCI = percutaneous coronary intervention; PPM = permanent pacemaker; SAVR = surgical aortic valve replacement; STS PROM = Society of Thoracic Surgeons score for predicted risk of operative mortality; TAVR = transcatheter aortic valve replacement; TMVR = transcatheter mitral valve replacement.

**Table 2:** Baseline Echocardiographic Data

LVEF	45.8 ± 12.3
LV ESD	4.7 ± 0.9
LV EDD	6.1 ± 0.7
Etiology of mitral valve dysfunction	
Degenerative	6 (25%)
Functional	16 (67%)
Mixed	2 (8%)
MR	
Moderate	0 (0%)
Moderate-severe	7 (29%)
Severe	17 (71%)
MVG	2.0 ± 1.0
Mitral leaflet tethering	8 (33%)
Anterior	1 (4%)
Posterior	3 (13%)
Bileaflet	4 (17%)
Mitral annular calcification	9 (38%)
Aortic stenosis	
Mild	0 (0%)
Moderate	2 (8%)
Severe	0 (0%)
Aortic regurgitation	
None/trivial	17 (71%)
Mild	7 (29%)
Moderate	0 (0%)
Severe	0 (0%)
Tricuspid regurgitation	
None/trivial	2 (8%)
Mild	5 (21%)
Moderate	10 (42%)
Severe	7 (29%)

Values are mean ± SD or n (%).

EDD = end-diastolic volume; ESD = end-systolic volume; LV = left ventricle; LVEF = left ventricular ejection fraction; MR = mitral regurgitation; MVG = mitral valve diastolic gradient.

### Procedural details

**Figure 1:**
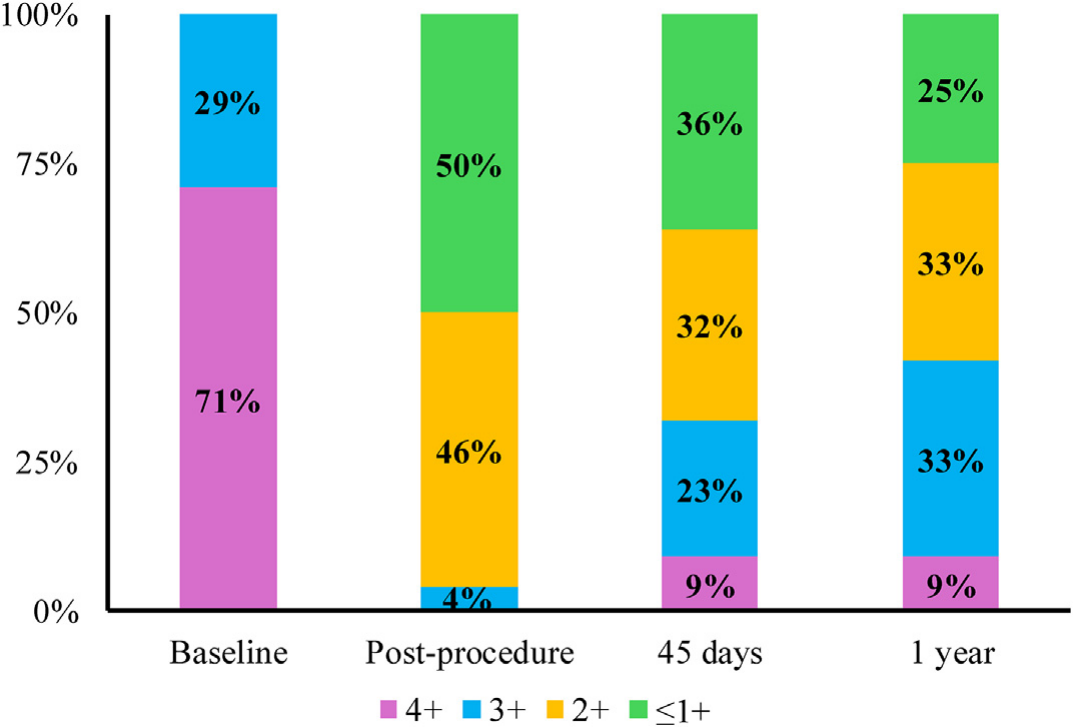
**The Change of Mitral Regurgitation at Follow-Up** The degree of mitral regurgitation in the included population at baseline, postprocedurally, and at 45 days and 1 year follow-up.

Total procedure time was 103.6 ± 33.7 minutes for both procedures. For the MTEER procedure, 1 device was deployed in 54% of patients, and the most common devices were MitraClip XTW and NTW (39% each). Residual MR of ≤2+ was achieved in all patients except one (Figure 1); this patient had 3+ MR after the placement of MitraClip NTW with a residual mitral valve diastolic gradient of 4 mm Hg, thus the placement of a second clip was unfeasible. The mean post-MTEER mitral valve gradient was 3.2 ± 1.6 mm Hg. The duration of the LAAO procedure was 22.1 ± 8.8 minutes. The most common device size used was the 35 mm (41.7%), and device margin leak was present in 2 patients with no leak >3 mm (Table 3).

**Table 3:** Procedural Characteristics

Total procedure time	103.6 ± 33.7
Fluoroscopy time	31.9 ± 18.4
Cumulative Air Kerma	754.2 ± 576.2
Dose Air Product	97.2 ± 72.7
Length of stay	2.8 ± 4.3
General anesthesia	25 (100%)
Transesophageal guidance	25 (100%)
MTEER procedure	
Procedure completed	24 (100%)
Duration of procedure	74 ± 29.4
No. of devices deployed	
1	13 (54%)
2	10 (42%)
3	1 (4%)
Type of devices deployed	
XT	3 (8%)
NT	5 (14%)
XTW	14 (39%)
NTW	14 (39%)
Residual MR	
≤1+	12 (50%)
2+	11 (46%)
3+	1 (4%)
4+	0 (0%)
Post-MTEER mitral valve gradient	3.2 ± 1.6
LAAO procedure	
Procedure completed	24 (100%)
Duration of procedure	22.1 ± 8.8
No. of devices deployed	
1	24 (100%)
Size of the WATCHMAN device	
24 mm	2 (8.3%)
27 mm	6 (25%)
31 mm	6 (25%)
35 mm	10 (41.7%)
Device margin leak present	2 (8.3%)
Device margin leak >3 mm	0 (0%)

Values are mean ± SD or n (%).

LAAO = left atrial appendage occlusion; MR = mitral regurgitation; MTEER = mitral transcatheter edge-to-edge repair.

### Procedural outcomes

Two patients experienced a procedural complication (Table 4). One patient developed a small pericardial effusion that did not require pericardiocentesis, and another patient developed an access site hematoma that resolved with manual compression. There was no procedural death or need for urgent surgery.

**Table 4:** Procedural Outcomes

Death	0 (0%)
Conversion to open-heart surgery	0 (0%)
Air embolism	(0%)
Myocardial infarction	(0%)
Pericardial effusion	1 (4%)
Pericardial effusion requiring pericardiocentesis	0 (%)
Esophageal injury	0 (0%)
New dialysis requirement	0 (0%)
Ischemic stroke/TIA	0 (0%)
Hemorrhagic stroke	0 (0%)
Access site hematoma	1 (4%)
Retroperitoneal bleeding	0 (0%)
Other hemorrhage	0 (0%)
Fistula	0 (0%)
Pseudoaneurysm	0 (0%)
MTEER single leaflet attachment	0 (0%)
Mitral leaflet injury	0 (0%)
Complete leaflet detachment	0 (0%)
Mitral subvalvular injury	0 (0%)
MTEER device embolization	0 (0%)
LAAO device migration	0 (0%)
LAAO device embolization	0 (0%)
ASD closure	3 (12%)

Values are n (%).

ASD = atrial septal defect; LAAO = left atrial appendage occlusion; MR = mitral regurgitation; MTEER = mitral transcatheter edge-to-edge repair; TEER = transcatheter edge-to-edge repair; TIA = transient ischemic attack.

### Clinical outcomes at 45-days

**Figure 2:**
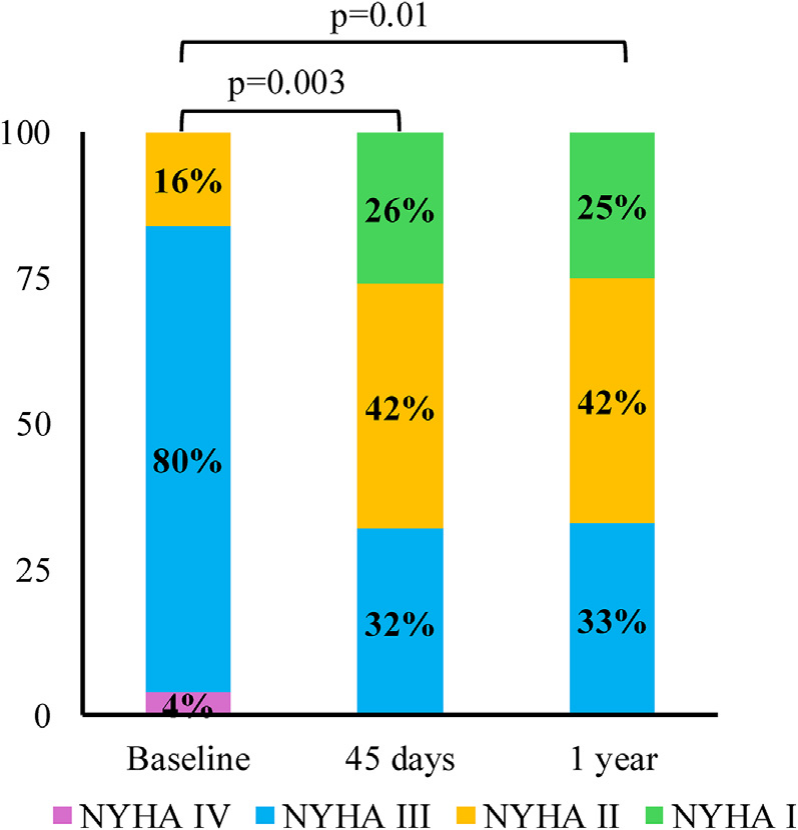
**Functional Capacity at Follow-Up** The change in functional capacity was assessed by NYHA functional class in the included population at baseline, at 45 days, and at 1-year follow-up.

**Figure 3:**
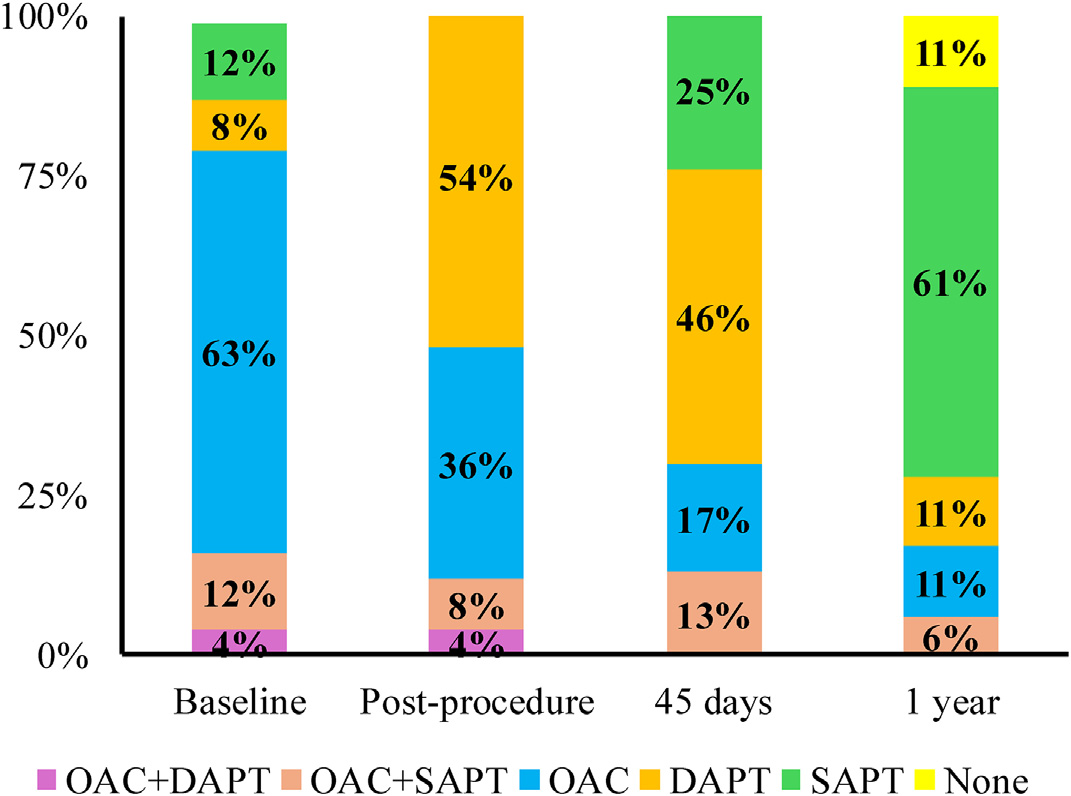
**Anticoagulation and Antiplatelet Regimen** The change in oral anticoagulation and antiplatelet regimen in the included population at baseline, postprocedurally, and at 45 days and 1 year follow-up. DAPT = dual antiplatelet therapy; OAC = oral anticoagulation; SAPT = single antiplatelet therapy.

Follow-up data at 45 days were available in all patients (Central Illustration and Table 5). The primary composite outcomes of all-cause mortality, stroke, or major bleeding occurred in 21% (95% CI: 5%-37%, n = 5/24) of patients; patient #1 experienced sudden cardiac death on day 34 after the procedure, patient #2 experienced an ischemic stroke on day 5 postprocedurally, TEE on admission showed well-seated WATCHMAN device with no DRT or peri-leaks, patient #3 experienced a traumatic head injury resulting in a hemorrhagic stroke on day 3 postprocedurally, patient #4 experienced an episode of hematuria that required hospitalization. Patient #5 was hospitalized with HF symptoms after 14 days postprocedurally. The patient received subcutaneous heparin for deep vein thrombosis prophylaxis, which results in significant bleeding and oozing at the injection site requiring multiple dressing changes and eventually a blood transfusion. Additionally, during this hospitalization, patient #5 was found to have severe MR and subsequently underwent surgical mitral valve replacement and was later discharged home. At 45 days, there was significant improvement in functional capacity at follow-up, with 68% had NYHA functional class I-II symptoms (Figure 2) and most patients were on DAPT (46%) (Table 6, Figure 3). Imaging data at 45 days showed residual MR ≤moderate in 68% and ≤mild in 36% (Figure 1, Table 7). DRT was detected in 1 patient, and the patient was transitioned from DAPT to apixaban. Peridevice leak >3 mm was not detected in any patient.

**Central Illustration:**
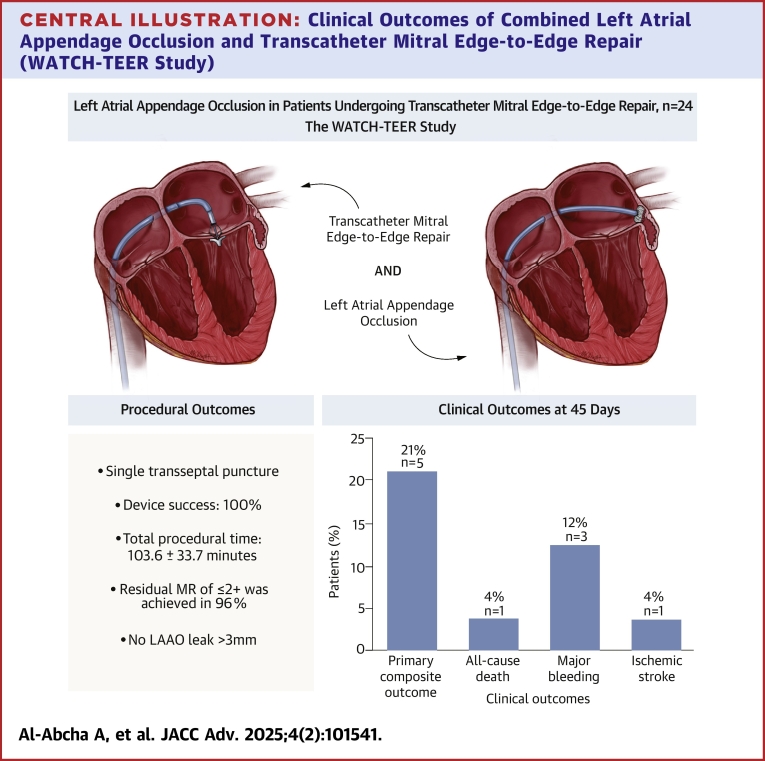
**Clinical Outcomes of Combined Left Atrial Appendage Occlusion and Transcatheter Mitral Edge-to-Edge Repair (WATCH-TEER Study)** Clinical outcomes at 45 days following the combined procedure of left atrial appendage occlusion (LAAO) and transcatheter mitral edge-to-edge repair (MTEER) in patients with indications for both treatments. MR = mitral regurgitation.

**Table 5:** Clinical Outcomes at 45 D and 1-Y Follow-Up

	0-45 D^a^ (n = 24)	45 D-1 Y (n = 19)	Cumulative Incidence Rate at 1 Y (n = 24)
Primary composite endpoint	5 (21%)	3 (16%)	8 (33%)
All-cause death	1 (4%)	1 (5%)	2 (8%)
Cardiac death	1 (4%)	1 (5%)	2 (8%)
Noncardiac death	0 (0%)	0 (0%)	0 (0%)
HF hospitalization	2 (8%)	4 (21%)	6 (25%)
Major bleeding	3 (12%)	3 (16%)	6 (25%)
Life-threatening bleeding	0 (0%)	0 (0%)	0 (0%)
Stroke/TIA	1 (4%)	0 (0%)	1 (4%)
Hemorrhagic stroke	1 (4%)	0 (0%)	1 (4%)
Major vascular complications	0 (0%)	0 (0%)	1 (4%)
Minor vascular complications	1 (4%)	0 (0%)	1 (4%)
Myocardial infarction	0 (0%)	1 (5%)	1 (4%)
New dialysis	1 (4%)	1 (5%)	2 (8%)
Endocarditis	0 (0%)	0 (0%)	0 (0%)
Mitral valve-related intervention	1 (4%)	0 (0%)	1 (4%)
Unplanned cardiac intervention or surgery, nonmitral	0 (0%)	1 (5%)	1 (4%)
MTEER embolization	0 (0%)	0 (0%)	0 (0%)
LAAO-related intervention	0 (0%)	0 (0%)	0 (0%)
LAAO thrombus	1 (4%)	1 (5%)^b^	2 (8%)
LAAO migration	0 (0%)	0 (0%)	0 (0%)
LAAO embolization	0 (0%)	0 (0%)	0 (0%)

Values are n (%).

HF = heart failure; LAAO = left atrial appendage occlusion; MR = mitral regurgitation; MTEER = mitral transcatheter edge-to-edge repair; TIA = transient ischemic attack.

a Including procedural outcomes.

b The same patient had LAAO thrombus recurrence at 1 y.

**Table 6:** Medication at Baseline and Follow-Up

	Baseline (n = 24)	Postprocedure (n = 24)	45 Days (n = 24)	1 Year (n = 18)
Aspirin	9 (38%)	16 (67%)	20 (83%)	14 (78%)
P2Y12 inhibitors	3 (13%)	14 (58%)	11 (46%)	2 (11%)
Warfarin	3 (13%)	3 (13%)	2 (8%)	0 (0%)
DOACs	16 (67%)	8 (33%)	5 (21%)	3 (16%)
No antiplatelet or OAC	0 (0%)	0 (0%)	0 (0%)	2 (11%)
ACEIs/ARBs	9 (36%)	8 (32%)	8 (33%)	4 (22%)
Beta-blockers	22 (92%)	20 (80%)	21 (88%)	16 (89%)
Loop diuretics	19 (79%)	20 (80%)	22 (92%)	16 (89%)

Values are n (%).

ACEI = angiotensin-converting enzyme inhibitor; ARB = angiotensin II receptor blockers; DOAC = direct oral anticoagulation; OAC = oral anticoagulation.

**Table 7:** Echocardiographic Data at Follow-Up

	Baseline (n = 24)	45 Days (n = 22)	1 Year (n = 12)
LVEF	45.8 ± 12.3	46.5 ± 15	47.8 ± 8.1
LV ESD	4.7 ± 0.9	4.3 ± 1.1	3.6 ± 1.0
LV EDD	6.1 ± 0.7	5.7 ± 0.8	5.2 ± 0.6
MR			
Trivial/1+	0 (0%)	8 (36%)	3 (25%)
2+	0 (0%)	7 (32%)	4 (33%)
3+	7 (29%)	5 (23%)	4 (33%)
4+	17 (71%)	2 (9%)	1 (9%)
MVG	2.0 ± 1.0	4.7 ± 2.0	4.1 ± 1.6
TR			
Trivial	2 (8%)	2 (9%)	2 (16.7%)
Mild	5 (21%)	6 (26%)	2 (16.7%)
Moderate	10 (42%)	9 (39%)	3 (25%)
Severe	7 (29%)	5 (22%)	5 (41.7%)
RVSP	49.5 ± 9.6	48.2 ± 9.7	48.1 ± 14.6

Values are mean ± SD or n (%).

EDD = end-diastolic volume; ESD = end-systolic volume; LV = left ventricle; LVEF = left ventricular ejection fraction; MR = mitral regurgitation; MVG = mitral valve diastolic gradient; RVSP = right ventricular systolic pressure; TR = tricuspid regurgitation.

### Clinical outcomes through 1 year

Follow-up data at 1 year were available in 19 patients (Table 5). The primary composite outcomes of all-cause mortality, stroke, or major bleeding occurred in 16% (95% CI: 0%-32%, n = 3/19) of patients. Patient #6 experienced an episode of gastrointestinal bleeding at 7 months after the index procedure that required endoscopic clipping. Later, the patient presented with a fatal myocardial infarction complicated with incessant ventricular tachycardia and shock at 12 months. Patient #7 experienced gross hematuria after traumatic Foley catheter insertion that resolved with conservative management, and patient #8 experienced a spontaneous gross hematuria and was later diagnosed with bladder cancer. Although residual MR was ≤2+ in 58%, there was a persistent improvement in functional capacity with 67% remaining in NYHA functional class I-II symptoms at 1 year (Figure 2). In addition, 83% of patients were not on OAC at 1 year (Table 6, Figure 3).

## Discussion

This is the first study to prospectively investigate the feasibility and safety of combining MTEER with LAAO in patients who have an indication for both procedures. The study’s main findings are: 1) combining MTEER and LAAO is feasible with ∼20 minutes added to overall procedural time; 2) combining MTEER and LAAO appears to be safe with no patients experiencing procedure-related death, major vascular complications, conversion to open surgery, or ischemic stroke during the procedure; 3) most patients experienced significant improvement in functional capacity and were able to be off OAC during follow-up; 4) the composite safety events at 45 days were mostly nonprocedural-related suggesting the need for careful risk stratification in this high-risk population.

Our results provide important insights into clinical events rates in this patient population. The included cohort represents an extremely high-risk population with a median STS score of 12.9% (5.39-15.62) and an expected high risk of clinical events including mortality. However, the cumulative rate of all-cause mortality at 1 year was 8% (95% CI: 0%-19%, n = 2/24), which is lower than anticipated. Recent data from the STS – American College of Cardiology (ACC) TVT (Transcatheter Valve Therapies) registry have reported a mortality rate of 22% at 1-year in all-comers patients who underwent isolated MTEER.^21^ Furthermore, the rate of mortality in our cohort is similar to recent reports of all-comers undergoing isolated LAAO.^22^ These findings are reassuring that the combined procedure is feasible in terms of mortality in this selected cohort of extremely high-risk patients; however, direct comparison to patients undergoing isolated LAAO or MTEER is limited given the different patient characteristics and overall patient risks.

Additionally, our study also confirmed the safety of the combined procedure. Overall procedural adverse events were rare. One patient developed a small pericardial effusion that did not require pericardiocentesis, and another patient developed an access site hematoma that resolved with manual compression. This low safety event rate compares favorably with a prior retrospective event rate reported by D'Amico et al in 30 patients who underwent combined LAAO and MTEER.^23^ The difference can reflect the overall improved LAAO procedural experience and safety.^24^ Thus, the addition of LAAO to patients undergoing MTEER did not alter procedural safety in this cohort.

The primary composite outcomes of all-cause mortality, stroke, or major bleeding at 45 days occurred in 21% (95% CI: 5%-37%, n = 5/24) of patients, which deserves further scrutiny. A detailed review of these events suggests that their etiology was likely related to the baseline risk profile of the patients enrolled rather than the procedure itself. There was 1 sudden cardiac death that occurred on day 34 after the procedure in a patient with advanced heart failure who was reported to be at baseline health prior to this event. One ischemic stroke occurred on day 5 postprocedurally and TEE showed a well-seated WATCHMAN device with no DRT or peri-leaks. A hemorrhagic stroke occurred after a traumatic head injury on day 3 postprocedurally. Two additional nonprocedure-related major bleeding events: the first event is an episode of hematuria that required hospitalization, and the other patient received subcutaneous heparin injection for deep vein thrombosis prophylaxis that resulted in significant bleeding and oozing at the injection site, eventually requiring a blood transfusion. This rate of adverse events in the short term after the procedure is comparable to previous reports of combined MTEER and LAAO.^20^^,^^23^

Furthermore, recent reports have estimated the risk of major bleeding to be ∼10% at 1 year post-LAAO.^25^ Despite the majority of patients being off anticoagulation, the rate of major bleeding was higher in our cohort with a cumulative incidence rate of 25% (95% CI 8%-42%, n = 6/24) at 1 year. This is higher than expected even among this high-risk population. Importantly, all bleeding events were not procedure-related and likely reflect the unique risk profile of patients needing combined MTEER-LAAO. Nonetheless, these findings highlight the value of a randomized clinical trial to assess clinical events in those who undergo combined MTEER-LAAO compared to those with similar profile treated conservatively.

Key technical considerations for the combined MTEER-LAAO procedure included the number of venous and transseptal accesses required, the influence of transseptal puncture location on procedural success, and the potential for extended procedure and anesthesia duration.^26^, ^27^, ^28^, ^29^ Our data confirmed that these factors posed no major concerns. First, a single venous access were utilized for the combined procedure in all patients with no impact on access-site bleeding. Second, although transseptal access was obtained to optimize the MTEER procedure (typically in the mid-superior and posterior parts of the septum), the success rate in implanting the LAAO with no peri-device leak >3 mm was 100%. Third, MTEER time (74 ± 29.4 minutes) was similar compared to contemporary MTEER studies including the EXPAND G4 study (77.0 [56.0-104.0] minutes)^30^^,^^31^ and LAAO added only 22.1 ± 8.8 minutes to the total procedural time. Fourth, LAAO was performed after MTEER and given that the septal access size required for MTEER is larger, adding the LAAO procedure did not impact the size of the iatrogenic atrial septal defect.

The WATCH-TEER study demonstrates the feasibility of combined MTEER and LAAO. However, the efficacy of the combined procedures will require further investigation in large-scale studies. Unfortunately, a study of this nature is unlikely due to the unique risk profile of this patient population, the large sample size required, and the associated costs. In the interim, physicians considering a combined MTEER and LAAO can use these feasibility data to support their shared decision-making. Another major challenge would be reimbursement. Traditionally, payers have limited their reimbursement of transcatheter structural interventions to a single procedure even if 2 or more procedures were performed at the same time. Nonetheless, the emerging results of the WATCH-TAVR and OPTION trials are anticipated to potentially change this paradigm and open the door for reimbursement of combined procedures.^11^^,^^12^

### Study Limitations

Our study has several limitations. First, this is an early single-arm feasibility study with a small sample size. Additional larger studies are needed to confirm its findings. Second, we enrolled patients who have high-prohibitive surgical risk, which does not reflect the current national practice with MTEER. Hence, major bleeding event rates were higher than what is reported in contemporary registries. However, the primary efficacy metric of LAAO seems to be accomplished with no ischemic stroke recorded between 45 days and 1 year in this high-risk cohort. Third, we do not have a comparative arm of patients who were treated with MTEER and LAAO in separate settings. This could be explored in the future but was beyond the scope of this feasibility study. Fourth, given the exploratory nature of this study, there was no core-lab to adjudicate imaging findings. However, the clinical events were carefully adjudicated by the study PIs. Fifth, 1-year follow-up data is incomplete in 4 patients who were not due for follow-up yet. Hence, the 1-year results should be interpreted within the context of this limitation. Sixth, some patients were discharged on DAPT prior to the U.S. Food and Drug Administration's approval for DAPT as an alternative therapy for post-LAAO antithrombotic regimen, which is considered an off-label use. Seventh, the durability of MR reduction was lower than anticipated. This can be related to the small sample size; however, this needs to be further investigated in larger studies.

## Conclusions

Combining LAAO with MTEER in patients who have a clinical indication for both procedures is feasible. Larger studies with longer-term follow-up are needed to discern the incremental clinical benefit of this approach and identify the best candidates for a combined procedure.

Perspectives **COMPETENCY IN MEDICAL KNOWLEDGE:** The WATCH-TEER study demonstrates that combining MTEER with LAAO is feasible and can be performed with acceptable safety in patients who have clinical indications for both procedures.

**TRANSLATIONAL OUTLOOK:** Future research should focus on validating these findings in larger, randomized clinical trials to further define the role of this approach in clinical practice.

## Funding support and author disclosures

The study was sponsored by a research grant from 10.13039/100008497Boston Scientific. Dr Alkhouli is on the advisory board of Boston Scientific and Abbott. All other authors have reported that they have no relationships relevant to the contents of this paper to disclose.
