# Supplementary material for: Outcomes of Combined Left Atrial Appendage Occlusion and Transcatheter Mitral Edge-to-Edge Repair: The WATCH-TEER Study
Source: JACC Adv. 2025 Jan 8;4(2):101541. doi: 10.1016/j.jacadv.2024.101541 (PMC11760823; doi:10.1016/j.jacadv.2024.101541)
Supplement: Supplemental Material [file mmc1.docx]

**Outcomes of Combined Left Atrial Appendage Occlusion and Transcatheter Mitral Edge-to-Edge Repair (WATCH-TEER Study)**

| **Supplemental Table 1.** Subject Inclusion and Exclusion Criteria |
| --- |
| ***Inclusion Criteria*** |
| - Men and Women ≥ 18 years of age |
| - The patient has severe symptomatic mitral regurgitation meet criteria for the commercially available MitraClip |
| - The patient also has documented paroxysmal, persistent, or permanent atrial fibrillation AND The patient meets the WATCHMAN FLX labeling guidelines |
| - The patient is eligible for short-term oral anticoagulation therapy with Warfarin or a direct oral anticoagulant |
| - The patient or legal representative is able to understand and willing to provide written informed consent to participate in the trial |
| - The patient is able and willing to return for required follow-up visits |
| ***Exclusion Criteria*** |
| - Mitral valve anatomy not deemed suitable for TEER |
| - Moderate to severe mitral stenosis (mean gradient >10 mmHg or MVA <1.5 cm2) |
| - Contraindication for short-term anticoagulation |
| - The patient has intra-cardiac thrombus as visualized by TEE within 1 week prior to Watchman FLX procedure |
| - LAAO is deferred intra-procedurally |
| - Prior occlusion of LAA |
| - Implanted mechanical mitral valve |
| - The patient requires long-term warfarin therapy due to:   a. Secondary to conditions such as prior arterial embolism or other indications such as pulmonary embolism or deep vein thrombosis within the previous 6 months.  b. The patient is in a hypercoagulable state.  Exclude the patient if per medical record documentation the patient meets any of the following criteria: • Thrombosis occurring at under 40 years age • Idiopathic or recurrent VTE (venous thromboembolism • Thrombosis at an unusual site (cerebral veins, hepatic veins, renal veins, IVC, mesenteric veins) • Family history of VTE or of inherited prothrombotic disorder, recurrence/extension of thrombosis while adequately anti-coagulated |
| - The patient is actively enrolled in another trial of a cardiovascular device or an investigational drug (post-market study and registries are acceptable) |
| - The patient is pregnant, or pregnancy is planned during the course of the investigation if patient is of child-bearing potential |
| - Any clinically significant medical condition or presence of any laboratory abnormality performed prior to randomization that is considered by the investigator to be clinically important and could interfere with the conduct of the study or not meeting procedure guidelines for WATCHMAN FLX or TEER with the latest FDA approved generation MitraClip |
| - The patient has a life expectancy of less than one year |
